# Antimicrobial Properties of Aloe vera Ethanol Extract as a Denture Disinfectant: An In Vitro Study

**DOI:** 10.7759/cureus.59916

**Published:** 2024-05-08

**Authors:** Reem Hussen Salim, Alaa Mazza Salloum, Samar Ali Alsalameh, Mays Rezaa Khazem, Mohammad Y. Hajeer

**Affiliations:** 1 Department of Removable Prosthodontics, Faculty of Dentistry, University of Damascus, Damascus, SYR; 2 Department of Basic Science, Faculty of Dentistry, University of Damascus, Damascus, SYR; 3 Department of Pharmacognosy, Faculty of Pharmacology, University of Damascus, Damascus, SYR; 4 Department of Orthodontics, Faculty of Dentistry, University of Damascus, Damascus, SYR

**Keywords:** surface disinfectant, dentures stomatitis, staphylococcus aureus, candida albicans, aloe vera ethanolic extract

## Abstract

Introduction

The aim of this study was to evaluate the activity of an ethanolic extract of Aloe vera on *Candida albicans* and *Staphylococcus aureus.*

Materials and methods

A total of 42 heat-cured acrylic resin specimens were made and divided into three groups according to the disinfection method: (1) Corega disinfectant tablets; (2) ethanol extract of Aloe vera; and (3) distilled water (as a control group). Fresh Aloe vera whole leaves were washed with distilled water, chopped into small pieces, air-dried, and ground into powder. The powder was extracted with 95% ethanol. The acrylic specimens were contaminated with *C. albicans *and *S. aureus*, and then the specimens were immersed in study solutions for three minutes. The viable colonies were counted using the colony-forming units (CFU) method.

Results

The results showed a decrease in the number of *C. albicans* CFU for denture tablets and Aloe vera ethanoic extract groups compared to the negative control group. There were no significant statistical differences between the denture tablet group and the Aloe vera ethanolic extract group (P < 0.05). Aloe vera ethanolic extract groups significantly decreased the number of *S. aureus* CFU compared to the negative control group and less compared to the denture tablet, where significant statistical differences were found between the tablet group and the Aloe vera ethanolic extract group.

Conclusions

Within the limitations of this study, it was concluded that Aloe vera extract was effective against *C. albicans* and *S. aureus* when acrylic resin specimens were immersed for three minutes.

## Introduction

Denture stomatitis (DS) is a condition characterized by inflammation and tissue infection in the oral cavity. It appears as extended or limited redness and swelling in the region where the denture is placed. People who wear dentures have a 20-80% chance of developing DS, which can be attributed to several factors such as poor oral hygiene, ill-fitting prostheses, resin porosity, and the accumulation of bacterial plaque [1].

The development of DS can be attributed to various etiological factors, one of which is the presence of biofilm on the surface of the denture [2]. This biofilm is a complex mixture of pathogenic and opportunistic microorganisms, such as bacteria, fungi, desquamated epithelial cells, and biofilm matrix. The matrix of biofilms acts as a reservoir for oral bacteria like *Candida albicans*, which is thought to be the main causative factor in the pathophysiology of DS. It is noteworthy that *C. albicans* has a tendency to adhere to acrylic resins, and the anaerobic microenvironment between the denture and palatal mucosa further facilitates its growth. Moreover, the colonization of *Candida* is also attributed to co-aggregation with pre-attached microorganisms on the denture and oral mucosa, such as *Staphylococcus aureus*, which is known to have a bacterial-yeast interaction. In actuality, *S. aureus* and* C. albicans* are typically isolated from the dental prostheses of people with DS [3]. It is important to remember that *S. aureus* and* C. albicans* can colonize not only the surface that comes into contact with the oral mucosa but also the fissures and flaws in the material for dentures. As a result, dentures may eventually serve as a pathogen reservoir and a source of ongoing microbial exposure to the mucosa [4].

In this manner, denture cleansing is fundamental to avoid biofilm arrangement on the acrylic denture surface. Effective and comprehensive oral and prosthesis maintenance is of paramount significance in order to safeguard the well-being of the tissue and avert the development of infections associated with the utilization of dentures [5]. Denture disinfectants often contain alkaline peroxides. These chemical agents are made up of oxygen-releasing substances such as sodium percarbonate or perborate and alkaline detergents. Alkaline peroxides come in tablet or powder form and, when dissolved in a solution, transform into hydrogen peroxide. This type of peroxide is most effective against the organic deposits found in prosthetic limbs. However, regular use of these solutions may have drawbacks, including color changes and decreased acrylic resistance [6].

Medicinal plants represent a rich source of antimicrobial and antioxidant agents. Many of the natural products are lower cost and safer than other medications. Aloe vera products have been extensively utilized for their medicinal properties [7]. The dry regions of North America, Europe, and Asia are home to a diverse array of over 360 distinct species of aloes. Aloe vera, a perennial succulent plant, falls under the purview of the Aloeaceae family (a subfamily of the Asphodelaceae) [8]. Most studies have studied the activity of the gel individually or the activity of an outer layer. The aim of the study was to evaluate the activity of the whole Aloe vera leaf extract as a disinfectant solution for removable dentures.

## Materials and methods

Study settings

This study was conducted at the Department of Removable Prosthodontics, Faculty of Dentistry, University of Damascus. The protocol was approved by the University of Damascus’s Local Research Ethics Committee (approval number UDDS-2188-12112022/SRC-2890). Funding was obtained from the University of Damascus Scientific Research Funds (ref. no. 501100020595). The nature of this research work did not require registering this study in any clinical trial registry.

Sample size calculation

The sample size was calculated using the G*Power software (Version 3.1, Düsseldorf, Germany). The effect size was considered equal to 1.9, and the study’s power was set at 90%.

Specimen preparation

In total, 42 specimens (n = 7) were made of heat-cured acrylic resin BMS (BMS Dental, Capannoli, Italy). The specimens had dimensions of 10 × 10 × 3 mm [9]. They were divided into two main groups: *C. albicans* and *S. aureus*. Each main group was divided into three subgroups: (1) Corega disinfectant tablets; (2) ethanol extract of Aloe vera; and (3) distilled water (i.e., the negative control group).

Test organisms

*S. aureus* and *C. albicans *were clinically isolated from patients with DS in the Department of Removable Prosthodontics at Damascus University. Fungal swabs were taken from the mucous of the hard palate using sterile cotton swabs. The cotton swab was inserted into the mouth and gently pressed on the palate, then moved from front to back and back to front, rotating it several times and repeating the process for 30 seconds. Two swabs were taken from the patient. The first swab was placed in a tube containing BHI nutrient broth to isolate *S. aureus* and incubated in the incubator for 24 hours at 37 °C. The second swab was placed in a tube containing Sabouraud Dextrose Broth to isolate *C. albicans* and incubated in the fungal incubator for 48 hours at 27 ˚C.

Culture media

The culture media used was Sabouraud Dextrose Agar for *C. albicans* and mannitol salt agar for *S. aureus*. These media were prepared according to the manufacturer’s instructions. A particular weight of the powder was obtained by utilizing particular volumes of distilled water. The funnel-shaped carafes were appropriately halted with cotton fleece wrapped in aluminum thwart. The flask was, at that point, autoclaved to sterilize. It was at that point permitted to cool to almost 45-50 ˚C, sometime recently apportioning into the sterilized Petri dish and cleared out to gel on the level surface.

Preparation of plant extract

Fresh Aloe vera whole leaves were washed with distilled water, chopped into small pieces, air-dried until constant weight, and ground into powder. The extraction process was carried out by the maceration method, where 20 g of the plant powder was swaged into 200 mL of ethanol (95%) at room temperature with shaking for three days. In addition, an ultrasonic device was used. The process was repeated twice, then the extracts were filled, and the solvent was evaporated by a rotary evaporator [10]. The concentration of Aloe vera extract was based on previous studies, where the lowest concentration was taken. It was able to inhibit fungi and bacteria at 0.5 mg/mL [11].

Positive control

Denture tablets (Corega, GSK House, London, UK) were used in the positive control group. According to the manufacturer’s instructions, one Corega tablet was dissolved in 200 mL of distilled water [12], which was used as a disinfectant.

Antibacterial activities

The acrylic specimens were contaminated with *C. albicans* and *S. aureus *and incubated. They were then immersed in study solutions for three minutes. After that, each specimen was placed in a tube containing 1 mL of physiological serum, which was subjected to shaking action by an electric shaker. Moreover, 100 microliters of the previous serum were spread on an appropriate petri dish and incubated. The viable colonies were counted using the colony-forming units (CFU) method.

Statistical analysis

IBM SPSS Statistics for Windows, Version 27.0 (Released 2020; IBM Corp., Armonk, NY, USA) was used in the statistical analysis of the data. In each group, the mean and standard deviation values were calculated. For inferential statistics, the level of significance was set at 0.05. The Brown-Forsythe test was applied, and Games-Howell multiple comparison tests were employed.

## Results

**Figure 1:**
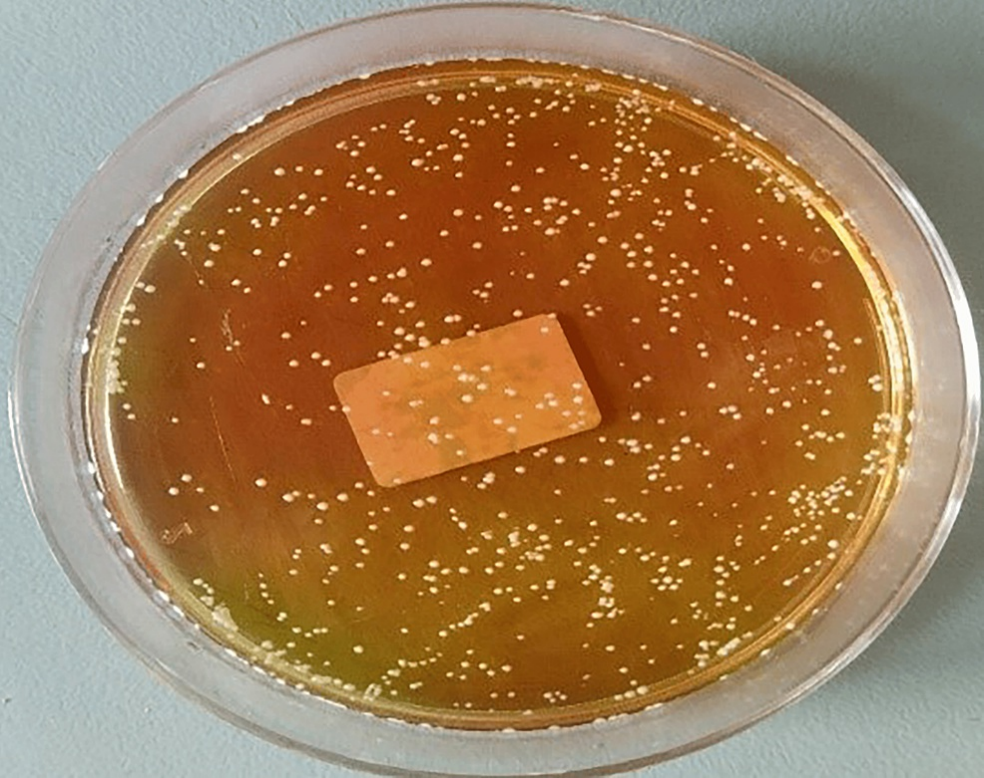
CFU/mL of Candida albicans in the negative control group CFU, colony-forming units

**Figure 2:**
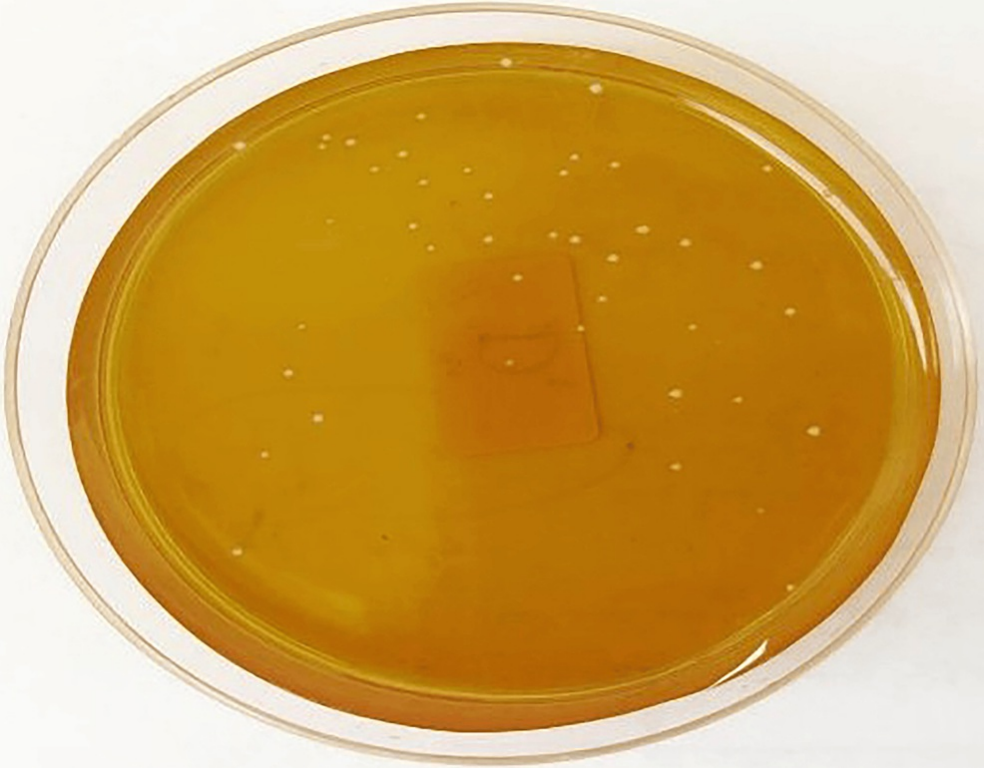
CFU/mL of Candida albicans in the Aloe vera group CFU, colony-forming units

Table 1 shows the lowest mean of *C. albicans* biofilm colony count was in the Corega group (3.314 CFU/mL) and the highest in the control group (4.414 CFU/mL). The mean values of the *C. albicans* biofilm colony count was lower in the Corega and ethanol extract of Aloe vera (3.614 CFU/mL) groups than in the control group (Figures 1, 2).

**Table 1:** Descriptive statistics of the CFU/mL in the C. albicans group CFU = number of colonies * dilution factors/volume of the culture plate CFU, colony-forming units

*Candida albicans *group	Mean (CFU/mL)	Standard deviation	Standard error of the mean	Minimum	Maximum
Control group	4.414	0.04	0.106	4.300	4.600
Corega group	3.314	0.14	0.3716	2.800	3.900
Ethanol extract of Aloe vera group	3.614	0.04	0.1069	3.500	3.800

Table 2 reveals the differences between the means of the control group and each of the other groups. There are statistically significant differences between the means of the control group and the other groups. There are no statistically significant differences between the means of Corega and ethanol extracts in the Aloe vera group.

**Table 2:** Comparisons between the three subgroups of the Candida albicans group using Games-Howell tests

Group I	Group J	Mean difference (I-J)	Standard error	Significance	95% confidence interval	
					Lower bound	Upper bound
Control group	Corega	1.19	0.10942	0.000	0.8338	1.5377
	Ethanol extract of Aloe vera	0.80	0.05714	0.000	0.6303	0.9697
Corega group	Ethanol extract of Aloe vera	-0.39	0.10942	0.256	-0.7377	-0.0338

**Figure 3:**
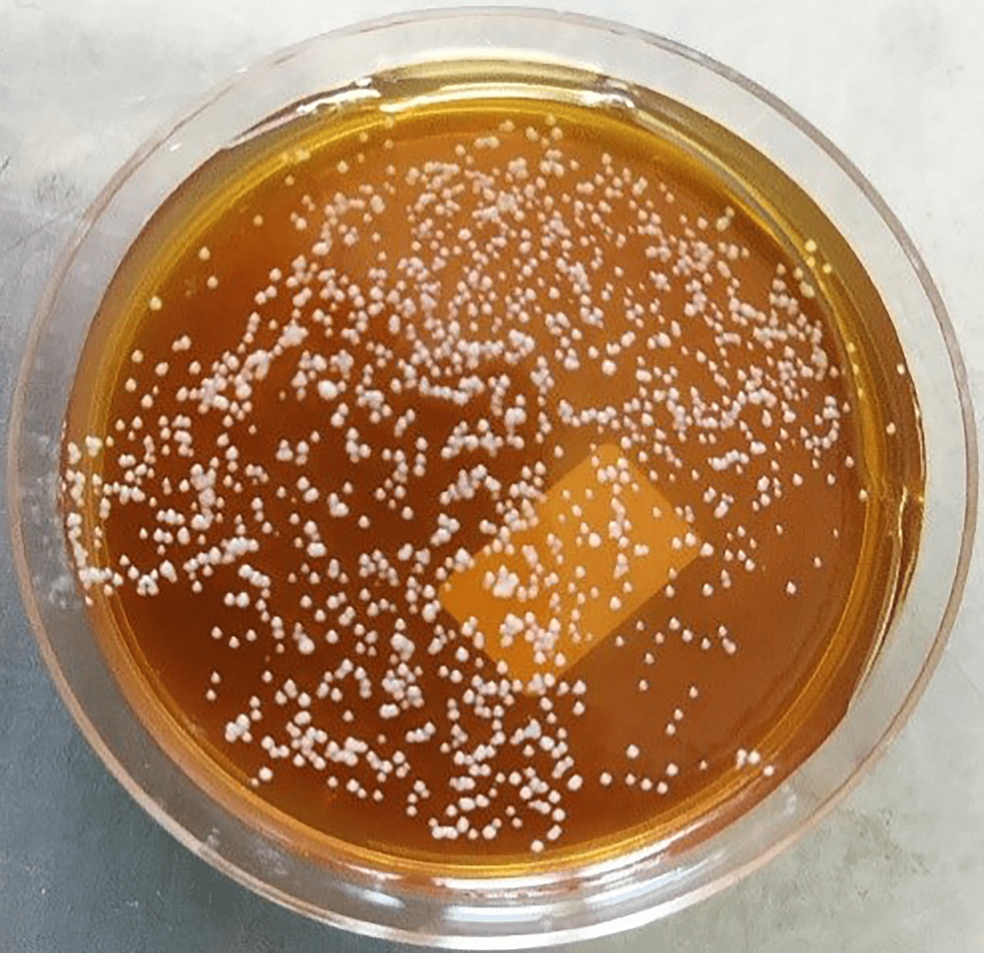
CFU/mL of Staphylococcus aureus in the negative control group CFU, colony-forming units

**Figure 4:**
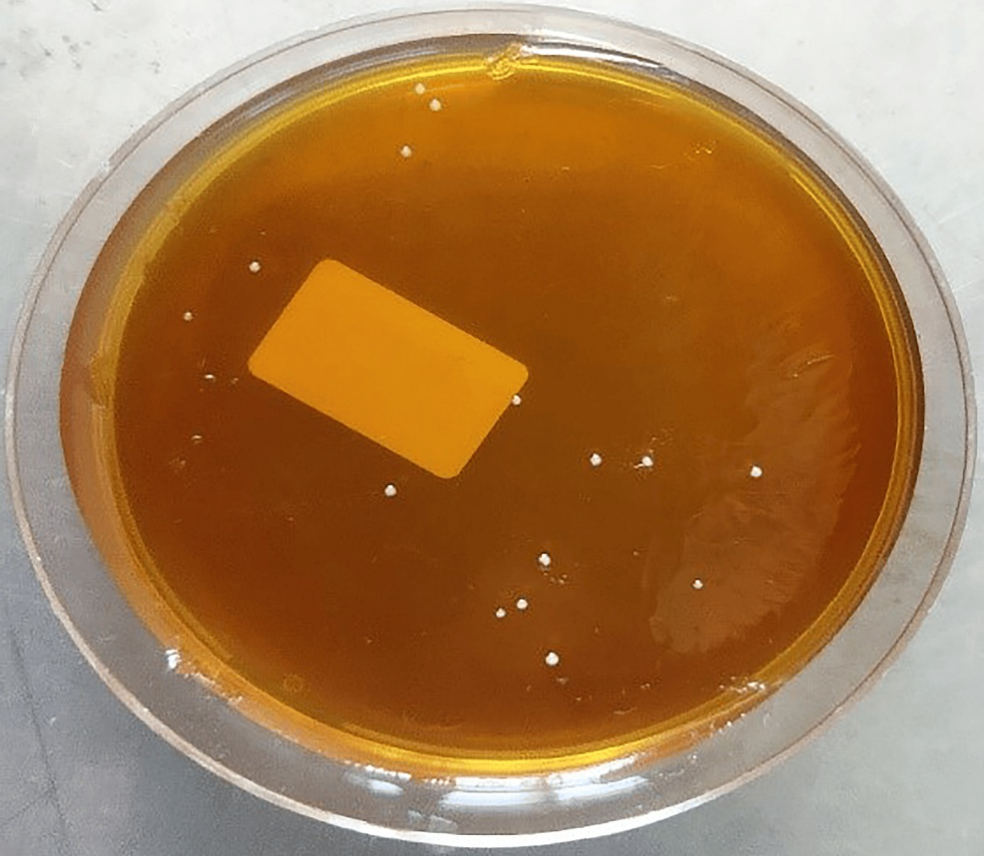
CFU/mL of Staphylococcus aureus in the Aloe vera group CFU, colony-forming units

Table 3 shows that the lowest mean of* S. aureus *biofilm colony count was in the Corega (0.385 CFU/mL) and the highest in the control group (4.443 CFU/mL). The mean *S. aureus* biofilm colony count for ethanol extract of Aloe vera (3.586 CFU/mL). The mean values of the* S. aureus* biofilm colony count were lower in the Corega and ethanol extracts in the Aloe vera groups than in the control group (Figures 3, 4).

**Table 3:** Descriptive statistics of the CFU/mL in the S. aureus group CFU, colony-forming units

*Staphylococcus aureus* group	Mean (CFU/mL)	Standard deviation	Standard error of the mean	Minimum	Maximum
Control group	4.443	0.08	0.02497	4.300	4.500
Corega group	0.385	0.66	0.2492	0.000	1.400
Ethanol extract of Aloe vera group	3.586	0.13	0.04738	3.400	3.700

Table 4 shows the differences between the means of the control group and each of the other groups. There are statistically significant differences between the means of the control group and the other groups. When studying the differences between the means of the disinfected Corega group and the Aloe vera ethanol extract group, the value of the significance level was less than 0.05. Therefore, it can be concluded that at the 95% confidence level, there were statistically significant differences between the mean values of the Corega and the ethanol extract of the Aloe vera group.

**Table 4:** Comparisons between the three subgroups in the Staphylococcus aureus group using Games-Howell tests

Group I	Group J	Mean difference (I-J)	Standard error	Significance	95% confidence interval	
					Lower bound	Upper bound
Control group	Corega	4.06	0.25098	0.000	3.1959	4.9184
	Ethanol extract of Aloe vera	0.71	0.05594	0.000	0.5434	0.8851
Corega group	Ethanol extract of Aloe vera	-3.34	0.25368	0.000	-4.2026	-2.4831

## Discussion

This in vitro study aimed to determine the effectiveness of Aloe vera against *C. albicans* and* S. aureus* and the ability to use Aloe vera extract as a denture disinfectant. Antifungal activity was tested for *C. albicans*, which is considered the main cause of DS. Antibacterial activity was also tested for *S. aureus*, which is considered one of the most common germs in patients who suffer from DS [13].

In this study, acrylic pieces were contaminated with both the fungi *C. albicans* and *S. aureus* separately, in a manner relatively similar to the biofilm formed on the surface of a denture. We took swabs from a patient with DS in order to select strains with high virulence and to mimic them as much as possible in clinical reality. The whole leaf of Aloe vera was studied to benefit from the compounds contained in the gel, such as polysaccharides, in addition to the chemical compounds of the outer layer, such as anthraquinones. The ethanol extract of the Aloe vera plant was used, which is considered more effective than the methanol extract [10].

The results of this study revealed that Aloe vera extract was effective against *C. albicans*, as it reduced the number of fungal colonies significantly in comparison with the control group. This may be attributed to the content of the aloe vera leaf. The content of Aloe vera leaf has many chemical components, including flavonoids, alkaloids, tannins, saponins, steroids, lactones, and anthraquinones, as these components play an important role in inhibiting the growth of *C. albicans* [14]. The results of the current study agree with the study of Nabila and Putra, which confirmed the effectiveness of the ethanol extract of Aloe vera against *C. albicans*, in which the ethanolic extract was studied on *Candida* from a laboratory and a disc diffusion method was used [15].

In addition, this study concluded that the ethanol extract of Aloe vera reduced the number of *S. aureus *colonies. The descriptive statistical study showed a decrease in the number of bacterial colonies by approximately 0.9 log cycles, a rate of 87%. Statistically significant differences were found between the ethanol extract of the Aloe vera group and the control group, and this confirms the effectiveness of the Aloe vera extract on *S. aureus*. The Aloe vera plant contained a high percentage of anthraquinone glycosidic compounds, imidine, and aloeimidine. These components work effectively to inhibit the formation of bacterial biofilms in addition to damaging the DNA of cells, leading to the death of the bacterial cell [16]. The results were consistent with the study of Arbab et al. in 2021, which demonstrated the effectiveness of Aloe vera gel in eliminating *S. aureus *bacteria [17].

Furthermore, validated study results, which support the widespread use of the plant, suggest that the plant extract may be a novel and effective source of antibacterial and antifungal activity that is safe, nontoxic, and less expensive than denture tablets. It is recommended that more research be conducted to examine and separate these chemicals, as well as analyze their principles and mechanisms of action. This includes conducting studies with different extracts or studying the effect of changing the duration of immersion in solutions.

Limitations

One limitation of this current study is that the in vitro study was conducted on a relatively small sample and evaluated only one type of bacteria (*S. aureus*) and fungi (*C. albicans*). Another limitation is the only use of a single concentration of the ethanolic extract of the whole leaf of the Aloe vera plant. In addition, the investigation was based on a single duration of immersion with evaluation at different times.

## Conclusions

This in vitro study demonstrated that an ethanol extract from the whole leaf of the Aloe vera plant was effective against *C. albicans* and has similar effectiveness to denture tablets against *C. albicans*, as there were no statistically significant differences between the two groups. In addition, the ethanol extract of the whole leaf of the Aloe vera plant was effective against *S. aureus* but less effective than denture tablets, as statistically significant differences were found between the two groups.
